# Prognostic Factors of Small Non-Functional Pancreatic Neuroendocrine Tumors and the Risk of Lymph Node Metastasis: A Population-Level Study

**DOI:** 10.3389/fendo.2022.907415

**Published:** 2022-07-06

**Authors:** Qingquan Tan, Xing Wang, Yichen Li, Yingyi Liu, Xubao Liu, Nengwen Ke

**Affiliations:** ^1^ Department of Pancreatic Surgery, West China Hospital, Sichuan University, Chengdu, China; ^2^ The First Clinical College, Chongqing Medical University, Chongqing, China

**Keywords:** small tumors, non-functional, pancreatic neuroendocrine tumors, lymph node metastasis, prognosis, SEER

## Abstract

**Background:**

Small non-functional neuroendocrine tumors (NF-PNETs) are a heterogeneous subset of tumors with controversy regarding their optimal management. We aimed to analyze the prognostic factors of patients with small NF-PNETs and create a risk score for lymph node metastasis (LNM).

**Methods:**

Data of 751 patients with NF-PNETs ≤ 2 cm were obtained from the Surveillance, Epidemiology, and End Results (SEER) database. Multivariate survival analysis was performed to analyze the prognostic factors. Logistic regression was used to identify risk factors for LNM.

**Results:**

Of the 751 patients, 99 (13.2%) were confirmed to have LNM. In multivariate survival analysis, LNM (hazard ratio [HR], 2.12; 95% CI, 1.04–4.32, p = 0.040) was independently associated with disease-specific survival. Logistic regression identified that tumor location in the head of the pancreas (odds ratio [OR], 4.33; 95% CI, 2.75–6.81; p < 0.001), size ≥ 1.5–2 cm (OR, 1.84; 95% CI, 1.17–2.87; p = 0.009), and grade III–IV (OR, 7.90; 95% CI, 1.79–34.90; p = 0.006) were independent risk factors of LNM. According to the OR value, the risk of LNM was scored as follows: a score of 1 for tumors located in the body/tail of the pancreas and 4 for those located in the head; a score of 1 for tumors <1 cm and 2 for those ≥1.5–2 cm; and a score of 1 for tumors with grade I–II and 8 for those with grade III–IV. Finally, the median score for this cohort was 4, with an interquartile range of 3–6. Therefore, patients were classified as three groups based on the risk score system: a total score of 1–3 for low risk, 4–6 for intermediate risk (OR, 2.98; 95% CI, 1.59–5.60; p = 0.001), and 7–14 for high risk (OR, 8.94; 95% CI, 4.50–17.7; p < 0.001), with an incidence of LNM 5.0%, 13.5%, and 31.8%, respectively (p < 0.001).

**Conclusion:**

Surgical resection with regional lymphadenectomy is recommended for small NF-PNETs with malignant potential of LNM. A risk score for LNM based on tumor grade, location, and size may preoperatively predict LNM of small NF-PNETs and guide clinical practice.

## Introduction

Pancreatic neuroendocrine tumors (PNETs) are a group of rare tumors with a variety of clinical manifestations and biological behaviors (1). PNETs can be divided into functional and non-functional tumors. Non-functional PNETs (NF-PNETs) are defined as those without a clinical symptom of hormone overproduction (2). In the past few decades, because of the widespread use of high revolution image technology and elevated attention from both clinicians and radiologists, there is an increased detection of NF-PNETs, especially for small NF-PNETs (≤2 cm) (3, 4).

Surgical resection is the mainstay treatment for PNETs; however, the management of small NF-PNETs remains controversial. Several studies have demonstrated the safety and feasibility of conservative management for asymptomatic sporadic small NF-PNETs (5–7). Considering the relatively high risk of morbidity and mortality in pancreatic surgery as well as the indolent course of small NF-PNETs, both National Comprehensive Cancer Network (NCCN) and European Neuroendocrine Tumor Society (ENETS) guidelines suggest that patients with an asymptomatic small tumor may be selectively observed (2, 8). Nevertheless, results from some surgical cohorts showed that 10%–15% of small NF-PNETs had the malignant potential with regional and distant metastases for which surgery is recommended (9–12). Moreover, although patients with small NF-PNETs generally have a good prognosis after surgery, 4%–6% of recurrences or tumor-related deaths have been observed (9, 13–15). Due to the overall rarity and heterogeneity, the prognostic factors of small NF-PNETs are not well defined.

Lymph node metastasis (LNM) has been proved to be a robust prognostic factor for PNETs. However, data regarding the prognostic value of lymph node status in small NF-PNETs were limited and with contrasting results. Vega et al. found that LNM was an independent predictor of poor disease-specific survival (DSS) and overall survival (9). Data from the National Cancer Database (NCDB) showed that LNM significantly decreased the disease-free survival (DFS) in patients with small NF-PNETs (mean survival: 115 vs. 95 months, p < 0.0001) (14). Conversely, in a European study with 210 resected small NF-PNETs, the presence of positive lymph nodes was not associated with DFS (15). In addition, the indication for regional lymphadenectomy for small NF-PNETs has also been debated. NCCN guidelines recommend that lymphadenectomy may be performed in tumors with a size >1 cm (8). According to the ENETS guideline, lymphadenectomy is confined within tumors larger than 2 cm (2). Recently, a multicenter study reported that patients with NF‐PNETs measuring 1.5–2.0 cm had a much higher risk of LNM than patients with tumors < 1.5 cm (17.9% vs. 8.7%, p = 0.013) for whom lymphadenectomy should be considered (13). To date, tumor size is a major determinant of lymphadenectomy. In terms of the present controversy, more factors are needed to predict LNM and help choose an appropriate strategy for these patients.

Therefore, the primary aim of this study was to analyze the prognostic factor in patients with small NF-PNETs without distant metastasis. The second aim was to explore the risk of LNM and create a risk score based on preoperative factors.

## Methods

### Study Population and Materials

**Figure 1:**
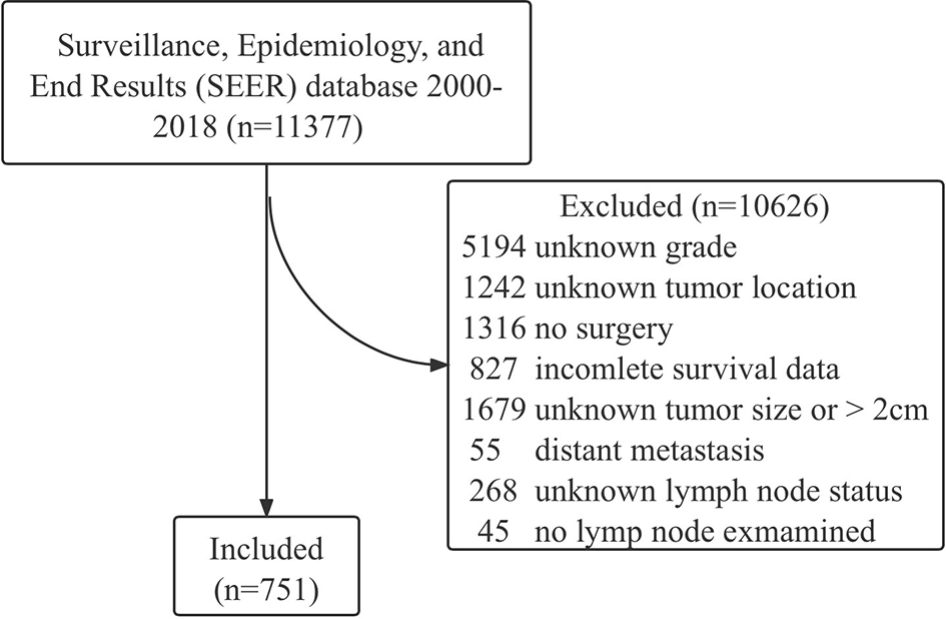
Flowchart of patient selection.

The data of patients with NF-PNETs were obtained from the Surveillance, Epidemiology, and End Results (SEER) database between 2000 and 2018 by SEER stat software (version 8.3.9), and the reference number was 18464-Nov2020. NF-PNETs were classified according to the International Classification of Diseases for Oncology, Third Edition (ICD-O-3) codes: 8150 (pancreatic endocrine tumor), 8240 (carcinoid tumor), 8241 (enterochromaffin cell carcinoid), 8242 (enterochromaffin-like cell tumor), 8243 (goblet cell carcinoid), 8246 (neuroendocrine carcinoma), and 8249 (atypical carcinoid tumor). The inclusion criteria were known grade, known location (head, body, tail), surgery performed, tumor size ≤2 cm, known lymph node status, at least one lymph node were examined, known death cause, and complete survival data. The exclusion criteria were synchronous distant metastasis (M1). The flowchart of patient selection is shown in **Figure 1**. Variables analyzed in this study included age, sex, race, year of diagnosis, tumor location, tumor grade (I, well differentiated; II, moderately differentiated; III, poorly differentiated; and IV, undifferentiated), tumor size, lymph node status, number of positive and examined lymph nodes, and type of surgery.

This international database study is exempt from institutional review board (local ethics committee of the Sichuan University, West China Hospital) approval.

### Statistical Analysis

Categorical variables were summarized using counts and percentages and compared using Pearson’s χ^2^ test or Fisher’s exact test, as appropriate. DSS was evaluated using the Kaplan–Meier method. Univariate survival analysis was performed by log-rank test. Multivariate survival analysis was conducted by Cox proportional hazards model, with results expressed as hazard ratio (HR) and 95% CI. Univariate and multivariate logistic regression analyses were used to identify preoperatively available factors associated with LNM, with results expressed as odds ratio (OR) and 95% CI. Based on the OR value of multivariate logistic regression analysis, a risk score for preoperatively predicting LNM was established. All statistical analyses were performed by SPSS 26.0 software (SPSS, Inc., Chicago, IL, USA) and GraphPad Prism (version 8.2.1). A p-value <0.05 was considered statistically significant.

## Results

### Patient Characteristics

In the SEER database, a total of 751 patients with small NF-PNETs without distant metastasis were identified for analysis (**Table 1**). There was no sexual difference, and most of the patients were white. Of these, 423 patients (56.3%) with age ≥ 60 years were classified as old people. The majority of patients (68.4%) had a tumor located in the body/tail of the pancreas. The median tumor size was 1.4 cm; thus, the cutoff value was defined as 1.5 cm. The most common surgery type was partial pancreatectomy (71%), followed by pancreaticoduodenectomy (17.3%), total pancreatectomy (8.1%), and local resection (3.6%). The percentages of patients with 1–5, 6–10, and ≥11 examined lymph nodes were 34.2%, 24.0%, and 41.8%, respectively. Most of the patients (95.8%) had a well or moderately differentiated (grade I–II) tumor, while 31 patients (4.1%) had a poorly differentiated or undifferentiated (grade III–IV) tumor.

**Table 1:** Baseline characteristics of small non-functional pancreatic neuroendocrine tumors.

Characteristics	Total, N (%)	Lymph node metastasis		p
		Presence, N (%)	Absence, N (%)	
Sex				0.501
Female	365 (48.6)	45 (45.4)	320 (49.1)	
Male	386 (51.4)	54 (54.6)	332 (50.9)	
Race				0.055
White	585 (77.9)	72 (72.7)	513 (78.7)	
Black	103 (13.7)	21 (21.2)	82 (12.6)	
Others	63 (8.3)	6 (6.1)	57 (8.7)	
Age, years				0.626
<60	328 (43.7)	41 (41.4)	287 (44.0)	
≥60	423 (56.3)	58 (58.6)	365 (56.0)	
Tumor location				<0.001
Head	237 (31.6)	61 (61.6)	176 (27.0)	
Body and tail	514 (68.4)	38 (38.3)	476 (73.0)	
Tumor size				0.020
<1.5 cm	393 (52.3)	41 (41.4)	352 (54.0)	
1.5–2 cm	358 (47.7)	58 (58.6)	300 (46.0)	
Type of resection				0.153
Local resection	27 (3.6)	6 (6.1)	21 (3.2)	
Formal resection	724 (96.4)	93 (93.9)	631 (96.8)	
Partial pancreatectomy	533 (71.0)	54 (54.5)	479 (73.5)	
Pancreaticoduodenectomy	130 (17.3)	28 (28.3)	102 (15.6)	
Total pancreatectomy	61 (8.1)	11 (11.1)	50 (7.7)	
Number of lymph nodes examined				0.006
1–5	257 (34.2)	22 (22.2)	235 (36.1)	
6–10	180 (24.0)	22 (22.2)	158 (24.2)	
≥11	314 (41.8)	55 (55.6)	259 (39.7)	
Grade				<0.001
I–II	720 (95.8)	72 (72.7)	648 (99.4)	
III–IV	31 (4.1)	27 (27.3)	4 (0.6)	

Of the 751 patients, 99 (13.2%) were confirmed to have LNM. Baseline characteristics for patients with and without LNM were compared (**Table 1**). The proportion of tumors located in the head of the pancreas was significantly higher in patients with LNM. Patients with LNM were more likely to have a large tumor size (p = 0.020), advanced tumor grade (p < 0.001), and large numbers of lymph nodes examined (p = 0.006). Patients with tumor size measuring 1.5–2 cm had a much higher prevalence of LNM compared with those with tumor size <1.5 cm (16.2% vs. 10.4%; p = 0.020). However, no difference was found in the incidence of LNM between patients with tumor size <1 and 1–1.5 cm (14/154, 9.1% vs. 27/239, 11.2%; p = 0.485).

### Survival Analysis

**Figure 2:**
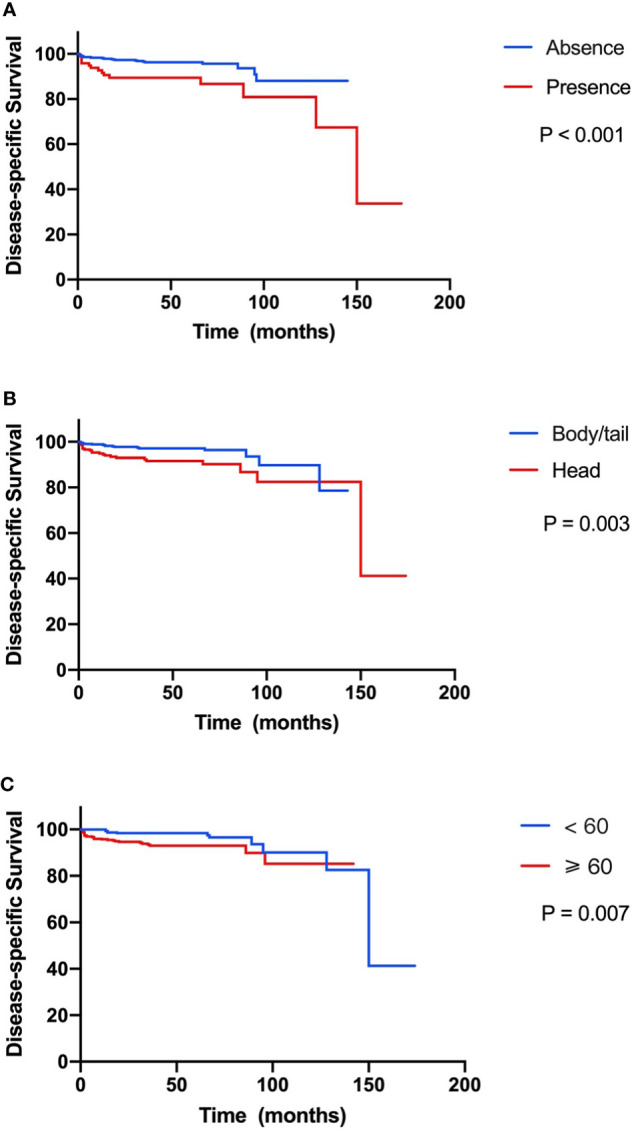
Kaplan–Meier curve of disease-specific survival stratified by prognostic factors. **(A)** Presence versus absence of lymph node metastasis. **(B)** Tumor located in head versus body/tail. **(C)** Age ≥60 versus <60 years.

In the 751 patients with small NF-PNETs, 41 disease-specific deaths (5.5%) were observed. The 1-, 5-, and 10-year DSS rates were 97.3%, 94.7%, and 87.1%, respectively. The 5-year DSS rate for patients with and without LNM was 86.7% and 93.4% (p < 0.001, **Figure 2A**). Moreover, tumor location in the head of the pancreas and old age (≥ 60 years) were associated with a poor DSS (p = 0.003 and p = 0.007, respectively; **Figures 2B, C**). Univariate survival analysis identified that sex, age, tumor location, number of lymph nodes examined, and LNM were associated with DSS (**Table 2**). In multivariate survival analysis, LNM (HR, 2.12; 95% CI, 1.04–4.32, p = 0.040) combined with age (HR, 2.43; 95% CI, 1.20–4.93; p = 0. 014) and tumor location (HR, 2.24; 95% CI, 1.15–4.35; p = 0.017) were independently associated with DSS in patients with small NF-PNETs.

**Table 2:** Univariate and multivariate analyses of factors associated with disease-specific survival.

Characteristics	Univariate analysis			Multivariate analysis		
	HR	95% CI	p	HR	95% CI	p
Sex, male	1.94	1.03–3.64	0.048	1.75	0.89–3.41	0.103
Race						
White		1				
Black	0.96	0.38–2.44	0.932			
Others	0.57	0.19–1.77	0.438			
Age, ≥60 years	2.47	1.32–4.62	0.007	2.43	1.20–4.93	0.014
Tumor location, head of pancreas	2.47	1.27–4.77	0.003	2.24	1.15–4.35	0.017
Tumor size, 1.5–2 cm	1.83	0.78–5.56	0.166			
Type of resection, formal resection	1.20	0.19–7.42	0.855			
Number of lymph nodes examined						
1–5		1				
6–10	1.75	0.62–4.94	0.271			
> 10	2.94	1.45–5.98	0.023	1.99	0.91–4.76	0.094
Lymph node metastasis, presence	2.70	1.18–6.22	<0.001	2.12	1.04–4.32	0.040
Grade, III–IV	3.90	0.40–38.3	0.243			

HR, hazard ratio.

### Risk Factors for Lymph Node Metastasis

**Figure 3:**
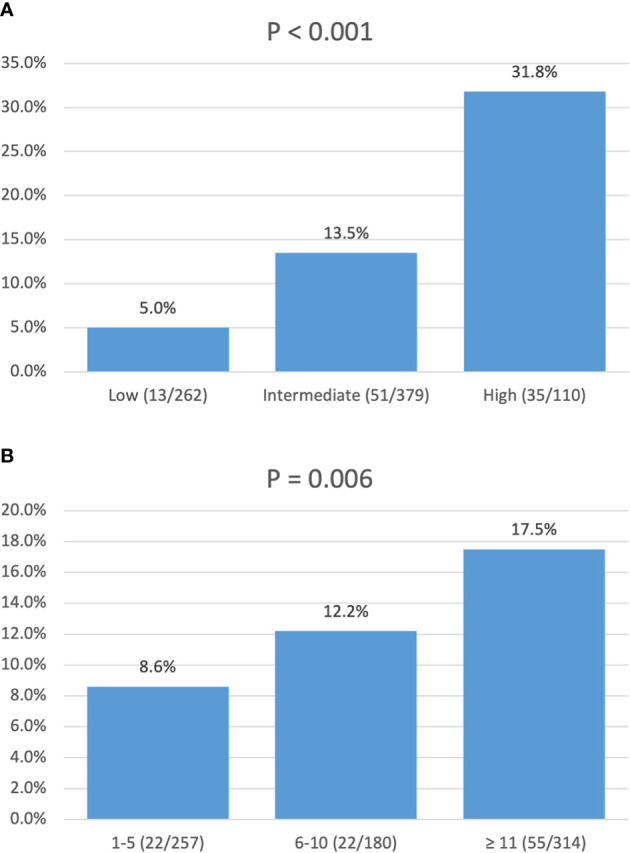
Incidence of lymph node metastasis: **(A)** stratified by risk score; **(B)** stratified by number of lymph nodes examined.

In a univariate logistic regression analysis (**Table 3**), factors including race, tumor location, tumor size, and tumor grade were associated with LNM. Multivariate analysis identified that tumor location in the head of the pancreas (OR, 4.33; 95% CI, 2.75–6.81; p < 0.001), size ≥ 1.5–2 cm (OR, 1.84; 95% CI, 1.17–2.87; p = 0.009), and grade III–IV (OR, 7.90; 95% CI, 1.79–34.90; p = 0.006) were independent risk factors of LNM. According to the OR value of multivariate analysis, the risk of LNM was scored as follows: a score of 1 for tumors located in the body/tail of the pancreas and 4 for those located in the head; a score of 1 for tumors < 1.5 cm and 2 for those ≥ 1.5–2 cm; and a score of 1 for tumors with grade I–II and 8 for those with grade III–IV. Finally, the median score for this cohort was 4, with an interquartile range of 3–6. Therefore, patients were classified into three groups based on the risk score system (**Table 4**): a total score of 1–3 for low risk, 4–6 for intermediate risk (OR, 2.98; 95% CI, 1.59–5.60; p = 0.001), and 7–14 for high risk (OR, 8.94; 95% CI, 4.50–17.7; p < 0.001). Patients in the high-risk and intermediate-risk groups were nearly 9 and 3 times more likely to develop LNM compared with those in the low-risk group (**Figure 3A**, p < 0.001). As shown in **Table 4**, the influence of the number of examined lymph nodes on LNM was also evaluated. Only patients with ≥11 examined lymph nodes were associated with LNM. For patients with 1–5, 6–10, and ≥11 examined lymph nodes, the incidence of LNM was 8.6%, 12.2%, and 17.5% (**Figure 3B**, p = 0.006), respectively.

**Table 3:** Logistic regression analysis of the risk factors for lymph node metastasis.

Characteristics	Univariate analysis			Multivariate analysis		
	OR	95% CI	p	OR	95% CI	p
Sex, male	1.12	0.72–1.71	0.627			
Race						
White		1			1	
Black	1.83	1.06–3.13	0.029	2.18	0.98–3.97	0.055
Others	0.75		0.520	0.86	0.34–2.15	0.746
Age, ≥60 years	1.16	0.76–1.77	0.502			
Tumor location, head of pancreas	4.32	2.80–6.74	<0.001	4.33	2.75–6.81	<0.001
Tumor size, 1.5–2 cm	1.66	1.08–2.55	0.02	1.84	1.17–2.87	0.009
Grade, III–IV	14.00	3.43–56.76	<0.001	7.90	1.79–34.90	0.006

OR, odds ratio.

**Table 4:** The risk for lymph node metastasis (LNM) stratified by risk score and number of examined lymph nodes.

Features	Number of patients	Events of LNM	Univariate logistic regression analysis		
			OR	95% CI	p
Risk score					
Low risk (1–3)	262	13		1	
Intermediate risk (4–6)	379	51	2.98	1.59–5.60	0.001
High risk (7–14)	110	35	8.94	4.50–17.77	<0.001
Number of examined lymph nodes					
1–5	257	22		1	
6–10	180	22	1.49	0.80–2.78	0.213
≥11	314	55	2.27	1.34–3.84	0.002

OR, odds ratio.

## Discussion

In this population-level study of 751 patients with resected NF-PNETs less than 2 cm, the overall 1-, 5-, and 10-year DSS rates were 97.3%, 94.7%, and 87.1%, respectively. The incidence of LNM was 13.2 (99/751). Multivariate survival analysis demonstrated that LNM combined with age and tumor location were independently associated with DSS. In a further logistic regression analysis, tumor location in the head of the pancreas, size 1.5–2 cm, and grade III–IV were independent risk factors for LNM. We further created a risk score for LNM of small NF-PNETs based on the OR values from logistic regression analysis and divided all patients into low-, intermediate-, and high-risk groups. The incidence of LNM in low-, intermediate-, and high-risk groups was 5.0%, 13.5%, and 31.8%, which was significantly different and increased with the risk level (**Figure 3A**, p < 0.001).

Small NF-PNETs are a heterogeneous subset of tumors with controversy regarding their optimal management. On the one hand, surgery has been considered a cornerstone for the management of NF-PNETs. A retrospective analysis from NCDB reported a significantly elevated 5-year overall survival of 82.2% in patients who underwent curative resection compared with a 5-year survival of 27.6% in patients who did not undergo surgery (11). Results from a meta-analysis also showed that an aggressive surgical policy for small NF-PNETs was associated with longer survival, while a watch-and-wait policy did not provide a benefit (16). On the other hand, in terms of the severe and relatively high incidence of complications in pancreatic surgery and the indolent course of small NF-PNET, conservative management has been recently proposed as a possible option. Two meta-analyses (5, 6) and several retrospective studies (7, 17, 18) have shown that a surveillance approach can be safely applied to selective patients with small asymptomatic NF-PNETs. Currently, the main conundrum for the management of small NF-PNETs is to identify patients with a high risk of malignancy.

The standard of selecting patients for surgery versus surveillance needs to be carefully evaluated. A retrospective study has demonstrated that small asymptomatic NF-PNETs have an unpredictable clinical course, and a subset of them may show aggressive behavior (12). A European multicenter study found that the presence of biliary or pancreatic duct dilatation and WHO grade 2–3 was associated with the recurrence of small NF-PNETs, and surgical resection was advocated in patients with these signs (15). In order to ensure the safety of conservative management, the NCCN guideline recommends that observation can be considered for small (<1 cm), low-grade, incidentally discovered tumors (8). Consistently, surveillance is preferred for low-grade, asymptomatic tumors with no suspicious malignancy in ENETS guidelines (2). In the present study, we found that lymph node status, age, and tumor location were independently associated with DSS in patients with small NF-PNETs, which implies that patients with a high risk of LNM, old age, and tumor located in the head of the pancreas may not be good candidates for observation. Nevertheless, for patients with old age or tumors located in the head of the pancreas, the morbidity of surgery should be taken into consideration. Enucleation (and regional lymphadenectomy), which has been proved to achieve the completable oncological outcomes compared with formal resections by previous studies (19–21) and our present data (p = 0.885), may be applied to these patients, when appropriate.

The reported incidence of LNM in small NF-PNETs varies from 2.6% to 27.5% (13–15, 22, 23). In our data, after the exclusion of patients with distant metastasis, the incidence of LNM was 9.9% in 751 patients with at least one examined lymph node. In line with most previous studies (9, 13, 14, 23), LNM was associated with the poor prognosis of small NF-PNETs in our study, suggesting the necessity of surgical resection and regional lymphadenectomy for patients with a high risk of LNM. Factors including age (9), tumor size (13), Ki-67 index (23), and lymphovascular invasion (14) have been considered predictors of LNM. In the present study, we focused on the factors that are available preoperatively. Of all the assessed factors, tumor grade III–IV was most highly associated with LNM (OR 7.90), although the proportion of these patients was relatively low. Tumor grade has been considered the main determinant of the malignancy of NF-PNETs, which is associated with not only the metastasis potential (9, 14) but also the long-term survival (15, 24). With the support of developed image technology, fine-needle aspiration biopsy (guided by endoscopic ultrasound (EUS), US, or CT) is recommended to evaluate the tumor grade preoperatively. Tumor location is another associated factor for LNM, with an OR of 4.33. Similar to our finding, Mei et al. found that tumors located in the pancreatic head were more likely to have LNM compared with those in the body/tail (42.8% vs. 30.9%, p < 0.001) and were associated with poor survival (25). The different embryological origins of the head and body/tail of the pancreas may partly contribute to these differences. As proved by previous studies (13, 14), tumor size was also related to the LNM of small NF-PNETs in this study. There was a significant difference in incidence of LNM (10.4% vs. 16.2%, p = 0.02) between tumors <1.5 cm and ≥1.5–2 cm. However, no difference was found in the incidence of LNM between patients with tumor size <1 and 1–1.5 cm. These results may give a reference to determine the therapeutic management of tumors between 1 and 2 cm. Based on three preoperatively available factors including tumor grade, location, and size, we created a risk score for LNM, which may be utilized to guide clinical practice and help choose an optimal strategy for the management of small NF-PNETs.

Although previous studies (26, 27) and our present data have shown that the detection rate of LNM is increasing with the number of examined lymph nodes, the minimal number of lymph nodes to be harvested for accurate nodal staging remains unclear. During pancreaticoduodenectomy, the number of harvested lymph nodes is generally adequate for an appropriate nodal staging. For distal pancreatectomy, Lopez-Aguiar et al. found that 7 or more lymph nodes should be examined for accurate staging (28), while a minimal number of 12 lymph nodes was suggested by Guarneri et al. (29). Moreover, the role of lymphadenectomy in organ-preserving surgeries such as enucleation and central pancreatectomy is undefined. In this study, we found that patients with ≥11 examined lymph nodes were more likely to have at least one positive node. However, more high-quality prospective studies are needed to determine the minimal number of lymph nodes in surgery for PNETs.

The strength of our findings is the population-level and long-term survival outcomes. However, there were several limitations in our study. The main limitations were the retrospective nature and the possible selection bias. All patients enrolled in this study have undergone surgery, which may lead to an overestimate of the malignant potential of small NF-PNETs. Furthermore, the SEER database does not collect information on recurrence; therefore, the primary end-point was DSS in our study. In addition, some tumor-related variables such as the Ki-67 index, vascular and perineural invasion, and postoperative complications are not available.

## Conclusion

Patients with small NF-PNETs have a favorable prognosis after surgery; however, a subset of them may show the malignant potential of LNM. Lymph node status combined with age and tumor location was associated with DSS in patients with small NF-PNETs. Surgical resection with regional lymphadenectomy is recommended for small NF-PNETs with malignant potential of LNM. A risk score for LNM based on tumor grade, location, and size may preoperatively predict LNM of small NF-PNETs and guide clinical practice.

## Data Availability Statement

The original contributions presented in the study are included in the article/**Supplementary Material**. Further inquiries can be directed to the corresponding authors.

## Ethics Statement

Ethical review and approval were not required for the study on human participants in accordance with the local legislation and institutional requirements. The ethics committee waived the requirement of written informed consent for participation.

## Author Contributions

QT: conception, data collection, manuscript drafting, and editing. XW: conception, data analysis, manuscript drafting, and editing. YCL: data collection and analysis, and manuscript editing. YYL: data collection and analysis, and manuscript editing. XL: resources, supervision, and manuscript review and editing. NK: conception, resources, and manuscript review and editing. All authors listed have made a substantial, direct, and intellectual contribution to the work and approved it for publication.

## Funding

This research was supported by Sichuan Province Science and Technology Planning Project (2020YFS0262), West China Hospital Clinical Research Incubation Project (21HXFH058), and the 1·3·5 Project for Disciplines of Excellence–Clinical Research Incubation Project of West China Hospital, Sichuan University (ZY2017302 and ZYJC21037).

## Conflict of Interest

The authors declare that the research was conducted in the absence of any commercial or financial relationships that could be construed as a potential conflict of interest.

## Publisher’s Note

All claims expressed in this article are solely those of the authors and do not necessarily represent those of their affiliated organizations, or those of the publisher, the editors and the reviewers. Any product that may be evaluated in this article, or claim that may be made by its manufacturer, is not guaranteed or endorsed by the publisher.
